# Complete genome sequence of Staphylococcus aureus, strain ILRI_Eymole1/1, isolated from a Kenyan dromedary camel

**DOI:** 10.1186/s40793-015-0098-6

**Published:** 2015-11-20

**Authors:** Saima Zubair, Anne Fischer, Anne Liljander, Jochen Meens, Jan Hegerman, Hadrien Gourlé, Richard P. Bishop, Ina Roebbelen, Mario Younan, Mudassir Imran Mustafa, Mamoona Mushtaq, Erik Bongcam-Rudloff, Joerg Jores

**Affiliations:** Department of Animal Breeding and Genetics, SLU Global Bioinformatics Centre, Swedish University of Agricultural Sciences, SE-75007 Uppsala, Sweden; International Livestock Research Institute, PO Box 30709, Nairobi, Kenya; International Center for Insect Physiology and Ecology, PO Box 30722, Nairobi, Kenya; Department of Infectious Diseases, Institute for Microbiology, University of Veterinary Medicine Hannover, Hannover, Germany; Institute of Functional and Applied Anatomy, Hannover Medical School, Hannover, Germany; Biomedical Research in Endstage and Obstructive Lung Disease Hannover (BREATH), Member of the German Center for Lung Research (DZL), Hannover, Germany; REBIRTH Cluster of Excellence, Hannover, Germany; Vétérinaires sans Frontières Germany, Nairobi, Kenya; Department of Public Health and Caring Science, Uppsala University, 751 22 Uppsala, Sweden

**Keywords:** *Staphylococcus aureus*, ST30, Camel, Pathogenicity islands, Core genome

## Abstract

We report the genome of a *Staphylococcus aureus* strain (ILRI_Eymole1/1) isolated from a nasal swab of a dromedary camel (*Camelus dromedarius*) in North Kenya. The complete genome sequence of this strain consists of a circular chromosome of 2,874,302 bp with a GC-content of 32.88 %. *In silico* annotation predicted 2755 protein-encoding genes and 76 non-coding genes. This isolate belongs to MLST sequence type 30 (ST30). Phylogenetic analysis based on a subset of 283 core genes revealed that it falls within the human clonal complex 30 (CC30) *S. aureus* isolate cluster but is genetically distinct. About 79 % of the protein encoding genes are part of the CC30 core genome (genes common to all CC30 *S. aureus* isolates), ~18 % were within the variable genome (shared among multiple but not all isolates) and ~ 3 % were found only in the genome of the camel isolate. Among the 85 isolate-specific genes, 79 were located within putative phages and pathogenicity islands. Protein encoding genes associated with bacterial adhesion, and secretory proteins that are essential components of the type VII secretion system were also identified. The complete genome sequence of *S. aureus* strain ILRI_Eymole1/1 has been deposited in the European Nucleotide Archive under the accession no LN626917.1.

**Electronic supplementary material:**

The online version of this article (doi:10.1186/s40793-015-0098-6) contains supplementary material, which is available to authorized users.

## Introduction

*S. aureus* is a bacterial species which has been isolated from diverse hosts including humans, other mammals and birds [1, 2]. In humans, it is persistently present in the nares of approximately 20 % of all individuals and intermittently carried by nearly 30 % individuals [3]. *S. aureus* has been reported to be a common cause of wound infections, pneumonia and bacteraemia in humans in Kenya [4, 5]. In small and large ruminants and pseudo ruminants such as dromedary camels (*Camelus dromedaries*), *S. aureus* causes mastitis and therefore negatively impacts the productivity of the dairy industry worldwide [6, 7].

Zoonotic transmission of *S. aureus* has also been reported [8, 9]. In arid and semi arid regions of the Greater Horn of Africa, camels represent an important and valuable livestock species that provides a significant percentage of the population with animal protein, particularly from milk [10]. Moreover, camel milk is often consumed raw without proper heat-treatment, which increases the risk of acquiring infections with zoonotic pathogens [11, 12].

Currently our knowledge of bacterial pathogens in camels is rather limited [13]. *S. aureus* has been reported to cause infections of the skin, udder, eyes and joints [14–17] in camels. In North Kenya between 1999 and 2004, the prevalence of *S. aureus* in camels has been reported as 54 % in closed skin abscesses, 36 % in open skin abscesses, 39 % in skin necrosis and 31 % in lymph node abscesses [15]. A recent survey reports the prevalence of intramammary infections (IMI) associated with *S. aureus* as 11 % in lactating camels in Kenya [16]. A study has also reported genotype data and identified ‘candidate’ virulence factors of *S. aureus* strains in Middle Eastern camels [14]. Here we present the complete genome sequence, annotation and comparative analysis of the *S. aureus* ST30 strain ILRI_Eymole1/1 isolated from a nasal swab of a dromedary camel in Kenya.

## Organism information

### Classification and features

**Fig. 1:**
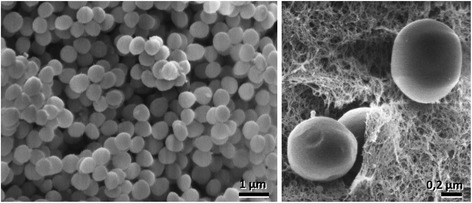
Scanning electron microscopy of ILRI Eymole 1/1 *S. aureus* grown on agar. Left: overview of cells grown in a colony; right: single cells in higher magnification

The *S. aureus* strain ILRI_Eymole1/1 was isolated in Kenya in 2004 from a nasal swab of a camel. It was identified as a member of the *Staphylococcus aureus* species on the basis of standard microbiological procedures [18] combined with a species-specific PCR [19]. *S. aureus* is a Gram-positive, coccus shaped, non-motile, nonspore forming and facultative anaerobic bacterium. *S. aureus* were grown on agar. Agar pieces were cut out and fixed in 150 mM HEPES, pH 7.35, containing 1.5 % formaldehyde and 1.5 % glutaraldehyde for 30 min at room temperature and at 4 °C over night. After dehydration in acetone and critical point drying, cells were gold sputtered and observed in a Philips SEM 505. Images were acquired using 10 kV at 10.000×/20 nm spot size or 40.00×/10 nm spot size. The bacterial cells are 0.5 to 1.0 mm in diameter, and occurs either singly or in the form of pairs or clusters (Fig. 1). The culture produces smooth, circular, glistening colonies of diameter > 5 mm. It produces a grey pigment. The general features of *S. aureus* strain Eymole1/1 are presented in Table 1 and Additional file 1: Table S1. The optimal growth temperature range is 37–42 °C. Tolerance to NaCl was tested in liquid medium, LB with NaCl concentrations between 0 and 4 M NaCl. Cells were grown overnight at 37 °C.

**Table 1:** Classification and general features of *Staphylococcus aureus* ILRI_Eymole1/1

MIGS ID	Property	Term	Evidence code^a^
	Classification	Domain *Bacteria*	TAS [49]
		Phylum *Firmicutes*	TAS [50, 51]
		Class *Bacilli*	TAS [52, 53]
		Order *Bacillales*	TAS [54–57]
		Family *Staphylococcaceae*	TAS [53, 58]
		Genus *Staphylococcus*	IDA
		Species *Staphylococcus aureus*	IDA
		Strain: ILRI_Eymole1/1 (Accession # LN626917.1)	
	Gram stain	Positive	IDA
	Cell shape	Grape-like coccus	IDA
	Motility	Nonmotile	IDA
	Sporulation	Nonspore-forming	IDA
	Temperature range	15–42 °C	IDA
	Optimum temperature	37 °C	IDA
	pH	4.5–9.5	IDA
	Optimum pH	7	
	Carbon Source	Glucose, fructose, mannose, maltose, lactose, trehalose, sucrose, turanose	IDA
MIGS-6	Habitat	Nasopharyngeal microflora	
MIGS-6.3	Salinity	1 to 2.5 M NaCl	IDA
MIGS-22	Oxygen requirement	Facultative anaerobe	TAS [59, 61]
MIGS-15	Biotic relationship	Free living	
MIGS-14	Pathogenicity	–	
MIGS-4	Geographic location	Kenya	
MIGS-5	Sample collection	01 February 2004	
MIGS-4.1	Latitude	3.916667	
MIGS-4.2	Longitude	41.833333	
MIGS-4.3	Depth	–	
MIGS-4.4	Altitude	220	

^a^Evidence codes - IDA: Inferred from Direct Assay; TAS: Traceable Author Statement (i.e., a direct report exists in the literature); NAS: Non-traceable Author Statement (i.e., not directly observed for the living, isolated sample, but based on a generally accepted property for the species, or anecdotal evidence). These evidence codes are from the Gene Ontology project [62]

Carbohydrate utilization was tested using ID 32 STAPH, a standardized system for the identification of the genera *Staphylococcus*, *Micrococcus* etc. (bioMérieux, Inc, Box 15969,Durham, NC 27704-0969 / USA). These tests showed positive results for glucose, fructose, mannose, maltose, lactose, trehalose, sucrose and turanose.

**Fig. 2:**
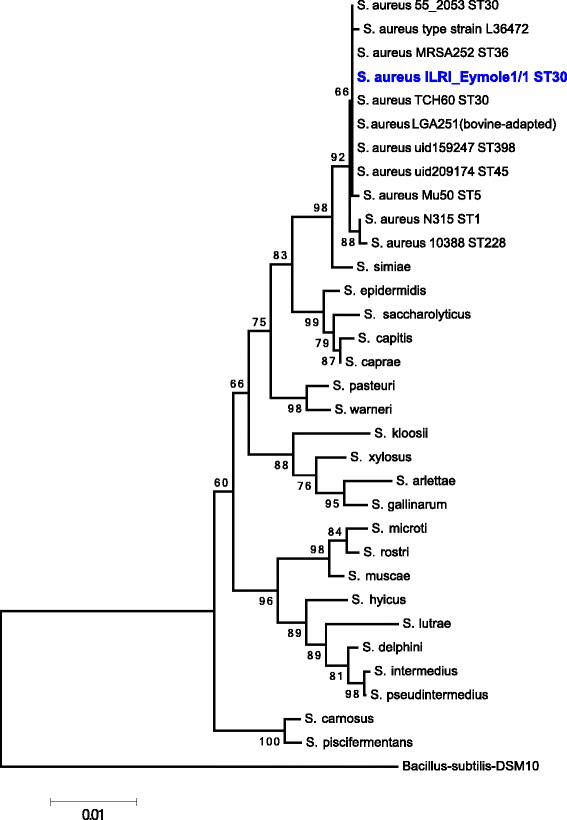
Phylogenetic tree showing the position of camel *S. aureus* strain ILRI_Eymole1/1 relative to other species of the genus *Staphylococcus* based on Muscle alignment of 1384 bp of 16S rRNA gene. The tree was constructed using MEGA v 6.06 [60, 63] implementing a Neighbor-Joining method with 1000 bootstrap replications and a Kimura 2-parameter model

The sequence type of the *S. aureus* isolate was determined using a previously described MLST dataset [20]. ILRI_Eymole1/1 belongs to ST30 MLST group. A BLASTn search [21] of all five copies of 16S rRNA sequence of ILRI_Eymole1/1 using default parameters revealed 99–100 % identity (with 98–100 % coverage) with all available *S. aureus* genomes in the database. The phylogenetic relationship was established using the 16S rRNA sequences of the type strains defining the genus *Staphylococcus* (accession numbers are provided in Additional file 2: Table S2). In addition, the 16S rRNA sequences of 9 *S. aureus* isolates (NC_021554, NC_017333, NC_017349, NC_022113, NC_002952, NC_017342, NC_002758, NC_002745, NC_020529) were extracted from the genome sequences, and a neighbor joining phylogenetic tree was constructed with MEGA v.6.06 (Fig. 2). The tree illustrates the close relationship of *S. aureus* ILRI_Eymole1/1 with *S. aureus* isolates from ST 30, 36, 5, 45 and the *S. aureus* type strain L36472 (Fig. 2). The position relative to other species within the genus *Staphylococcus* is also illustrated. *Bacillus subtilis* type strain DSM10 was used as an outgroup for the genus *Staphylococcus*.

## Genome sequencing information

### Genome project history

Twenty three strains of *S. aureus* have been isolated from healthy and diseased camels in East Africa using standard methods (1). The strains were isolated using primary cultivation on Columbia blood agar plates (Oxoid, UK) and were sub-cultured on mannitol-salt agar plates (Oxoid, UK). Afterwards the strains were subjected to multi locus sequence typing (2). Four strains belonged to sequence type 30, previously characterized in humans. The other isolates had novel sequence types that were likely to be camel specific. We selected the strain ILRI-Eymole1/1 for subsequent analysis since we wanted to elucidate the relationship of *S. aureus* isolates from camels and humans in order to project a zoonotic potential. S. aureus ILRI_Eymole1/1 was isolated from a nasal swab taken (transport Amies swab w/o charcoal, Copan, Italy) from a dromedary camel calf with rhinitis in Kenya in 2004.

### Growth conditions and genomic DNA preparation

The strain was grown in 10 ml liquid Brain heart medium (Carl Roth, Germany) at 37 °C and 200 rpm overnight. The strain was grown in 10 ml liquid Brain heart medium (Carl Roth, Germany) at 37 °C and 200 rpm overnight. The bacterial cells were pelleted using centrifugation at 5000 × g for 20 min. The supernatant was discarded and cells were subjected to genomic DNA isolation using the PureLink™ Genomic DNA Mini Kit (Invitrogen, USA) according to vendor’s instructions. The DNA was quantified using the Qubit® 3.0 Fluorometer (Thermo Scientific, Kenya) and the Qubit® dsDNA BR Assay Kit (Thermo Scientific, Kenya). The DNA concentration was 84.6 ng/ul, the 260/280 and 260/230 ratios were 1.49 and 0.56, respectively. To remove impurities, the DNA was further cleaned using a ratio of 1.6 AMPure beads (ref).

### Genome sequencing and assembly

Genome sequencing of *S. aureus* ILRI_Eymole1/1 was performed using the Illumina Genome Analyzer GAIIx platform. A 300 bp paired-end library with an average insert size of 550 bp was sequenced. The software MIRA v 4.0 [22] was used to assemble the *S. aureus* ILRI_Eymole1/1 genome, using as the input 1,154,246 Illumina paired-end reads. The *de novo* genome assembly generated a total of 118 contigs with average coverage of 109 × and average quality of 83 (Table 2). The whole genome alignment tools Mauve [23] and MUMmer v 3.2.2 [24] were used to order contigs of length greater than 1000 bp (69 contigs) against a reference genome sequence MRSA252/NC_002952 [25]. A complete genome sequence was obtained by joining the ordered contigs on the basis of their overlaps. The assembly output ACE file was viewed and analyzed in Tablet viewer version 1.13.05.17 [26].

**Table 2:** Project information

MIGS ID	Property	Term
MIGS-31	Finishing quality	Finished
MIGS-28	Libraries used	Illumina Paired End; Average read length 300 bp; Average insert size 550 bp.
MIGS-29	Sequencing platforms	Illumina GA-II
MIGS-31.2	Fold coverage	109 ×
MIGS-30	Assemblers	MIRA 4.0
MIGS-32	Gene calling method	RAST server, Basys
	Locus Tag	–
	Genbank ID	LN626917.1
	Genbank Date of Release	October 31, 2014
	GOLD ID	Gp0109422
	BIOPROJECT	PRJEB6577
MIGS-13	Source Material Identifier	ILRI_Azizi_biobank
	Project relevance	Bacterial pathogen in camels

### Genome annotation

The complete genome sequence of *S. aureus* ILRI_Eymole1/1 was annotated using RAST [27]. Ribosomal RNA genes were identified using RNAmmer server v 1.2 [28], and the tRNA genes were predicted using tRNAscan-SE v 1.21 [29]. The COG genes and associated functional categories information were downloaded from the COG database [30]. The COG categories were assigned to the ILRI_Eymole1/1 genome annotation using *blastp* v 2.2.28 [31] against the COG genes collection/ myva. Genes with more than 40 % amino acid identity and with e-values of less than 0.00005 were classified as putative homologues of genes within the COG database, and functional categories were assigned. Signal peptides were predicted using the SignalP v 4.1 server [32], and the transmembrane helices/ membrane spanning domains were identified using TopPred2 [33]. Phage-like sequences were predicted using PHAST [34].

## Genome properties

**Fig. 3:**
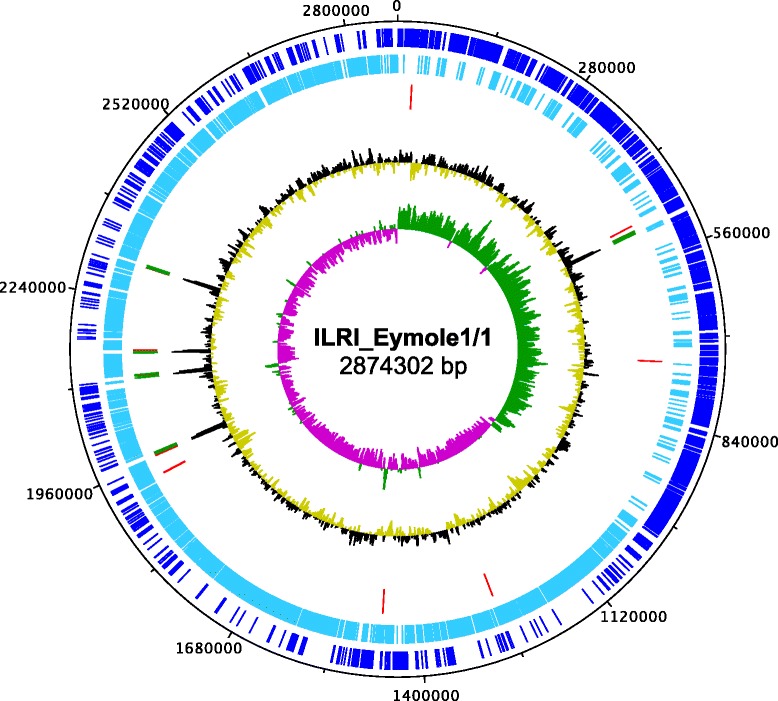
Circular map of the *S. aureus* ILRI_Eymole1/1 genome. From outer to inner circle; 1) Protein encoding genes in forward orientation are shown in dark blue, 2) Protein encoding genes in reverse orientation are shown in light blue, 3) Ribosomal RNAs are depicted in green while tRNAs are in red, 4) G + C-content plot and 5) GC-skew graph. The Graph was generated using DNAPlotter [64]

The *S. aureus* ILRI_Eymole1/1 genome is a circular chromosome of 2,874,302 bp with a GC-content of 32.88 %. A total of 2831 genes were predicted comprising 2755 protein encoding genes, 60 tRNA genes and 16 rRNA genes (Table 3, Fig. 3). Five copies of both 16S and 23S rRNA genes and six copies of 5S rRNA genes were identified. Among the predicted protein encoding genes, 652 (23.66 %) were hypothetical proteins. A total of 162 genes (5.88 %) were predicted to encode proteins with secretory signal peptides (potentially targeted to the secretory pathway) and 1040 (37.75 %) were genes encoding proteins with transmembrane helices or membrane spanning proteins. A total of 2054 (74.56 %) predicted genes were assigned to COG functional categories, while 701 (25.44 %) were not present within the COG collection (Table 4).

**Table 3:** Nucleotide content and gene count levels of the genome

Attribute	Value	% of total^a^
Genome Size (bp)	2,874,302	100.00
DNA coding	2,404,314	83.65
DNA G + C (bp)	945,066	32.88
Total genes	2831	100.00
Protein-coding genes	2755	97.32
RNA genes	76	2.68
Pseudo genes	0	0.00
Genes in internal clusters	n/a	n/a
Genes with function prediction	2170	76.65
Genes assigned to COGs	2054	74.56
Genes with Pfam domains	1688	59.63
Genes with signal peptides	162	5.88
Genes with transmembrane helices	1046	37.97
CRISPR repeats	0	0.00

^a^ The total is based on either the size of the genome in base pairs or the total number of protein coding genes in the annotated genome

**Table 4:** Number of genes associated with the 25 general COG functional categories

Code	Value	% of total^a^	Description
J	145	5.26	Translation
A	0	0.00	RNA processing and modification
K	120	4.36	Transcription
L	148	5.37	Replication, recombination and repair
B	0	0.00	Chromatin structure and dynamics
D	24	0.87	Cell cycle control, mitosis and meiosis
Y	0	0.00	Nuclear structure
V	45	1.63	Defense mechanisms
T	35	1.27	Signal transduction mechanisms
M	90	3.27	Cell wall/membrane biogenesis
N	1	0.04	Cell motility
Z	0	0.00	Cytoskeleton
W	0	0.00	Extracellular structures
U	20	0.73	Intracellular trafficking and secretion
O	61	2.21	Posttranslational modification, protein turnover, chaperones
C	91	3.30	Energy production and conversion
G	106	3.85	Carbohydrate transport and metabolism
E	174	6.32	Amino acid transport and metabolism
F	64	2.32	Nucleotide transport and metabolism
H	77	2.80	Coenzyme transport and metabolism
I	50	1.82	Lipid transport and metabolism
P	124	4.50	Inorganic ion transport and metabolism
Q	23	0.84	Secondary metabolites biosynthesis, transport and catabolism
R	228	8.28	General function prediction only
S	220	7.99	Function unknown
–	208	7.55	Other COG categories
–	701	25.44	Not in COGs

^a^The total is based on the total number of protein coding genes in the annotated genome

## Insights from the genome sequence

We performed a comparative analysis of the camel *S. aureus* ILRI Eymole1/1 isolate of sequence type 30 with 16 previously sequenced ST30 *S. aureus* isolates, two ST36 methicillin resistance *Staphylococcus aureus* isolates MRSA252, EMRSA16 and one ST431 *S. aureus* isolate M809, which together belong to the clonal complex 30 (CC30). ST36 and ST431 are single locus MLST variants of ST30. Previously sequenced *S. aureus* complete genome sequences were downloaded from the NCBI FTP site [35] (accession numbers are provided in Additional file 3: Table S3), and CC30 isolates were selected by analyzing their house keeping genes using the *S. aureus* MLST database [20]. The collection of draft *S. aureus* CC30 genomes was derived from previous studies [36, 37].

### Core genome analysis and COG classification

**Fig. 4:**
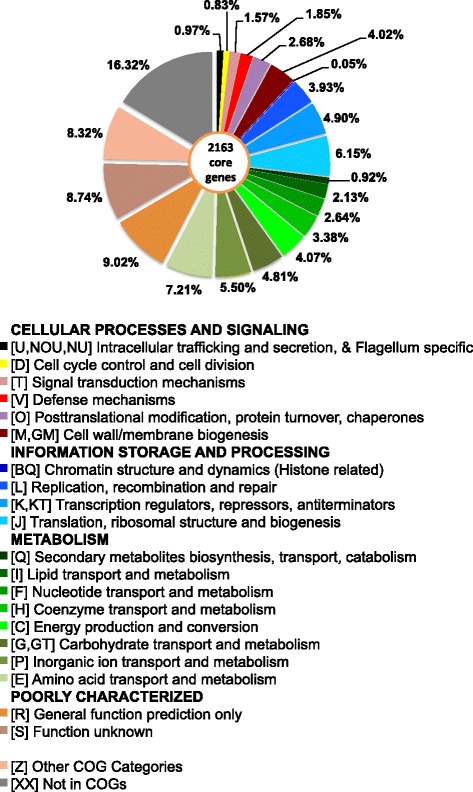
COG functional classification of CC30 *S. aureus* core genome

All 20 *S. aureus* CC30 genomes were annotated using the RAST server, and amino acid sequences of protein encoding genes from all CC30 genomes were used for the core genome analysis. Blastp searching of protein sequences of all CC30 isolates was carried out using local *blastp* v 2.2.28 [31]. The genes matching in all CC30 genomes with > =80 % identity, e-value < 0.00005, and alignment length > = 50 % were classified as core genes using custom scripts (Additional file 4: Supplementary material S4). The core genes were further analyzed for their COG functional classification using a matching criterion of > = 40 % identity and an e-value < 0.00005. Among 2163 core genes, 1810 (83.68 %) were present in COG database, whereas 353 (16.32 %) were not present in COG database. The functional classification of these genes is shown in Fig. 4.

*S. aureus* ILRI_Eymole1/1 variable genes (shared with some of the CC30 *S. aureus* genomes), and isolate-specific genes were also identified. We identified 2163 core genes (78.51 % of the total protein encoding genes), 507 (18.40 %) variable protein-encoding genes and 85 (3.09 %) isolate-specific genes.

### Bacterial adhesins

The colonization and adhesion of *S. aureus* to the nasal epithelial cells is thought to be mediated by surface proteins ClfB, IsdA and the serine-aspartic acid repeat proteins SdrC and SdrE. A published study demonstrated that a mutant lacking these four proteins did not exhibit the adherence phenotype [38]. *S. aureus* ILRI_Eymole1/1 possesses genes encoding fibrinogen-binding protein ClfB (CEH27447), adhesin proteins SdrC (CEH25318) and SdrE (CEH25319), in common with a subset of the CC30 *S. aureus* genomes. A gene encoding Heme/ Iron regulated surface protein IsdA (CEH26009) was present among the core protein repertoire of ILRI_Eymole1/1 isolate, and is known to be important for *S. aureus* infection of human skin, through mediating resistance to skin innate defense mechanisms [39].

*S. aureus* ILRI_Eymole1/1 possessed genes encoding many fibrinogen-binding proteins, including clumping factor/ fibrinogen binding protein ClfA, (CEH26520: variable gene), fibronectin/ fibrinogen binding protein FnBP (CEH25930: core gene), extracellular fibrinogen-binding protein Efb (CEH25981: core gene), fibronectin binding protein FnbB (CEH27312: variable gene), fibronectin binding protein FnbA (CEH27314: variable gene), and clumping factor ClfB, fibrinogen binding protein (CEH27447: variable gene). These FnBPs bind the host fibronectin receptor β1-integrins to promote *S. aureus* invasion of various mammalian cells including epithelial cells, endothelial cells and fibroblasts. These cells do not require specific co-receptors for *S. aureus* [40].

A gene encoding an extracellular adherence protein of broad specificity Eap/Map (CEH26760: core gene) was also identified in the camel *S. aureus* isolate. This protein has been reported to be involved in *S. aureus* internalization into the host cells. Eap is known to be responsible for agglutination of bacterial cells by rebinding to the surface of *S. aureus*. It shows dual affinity for the cell surface plasma proteins as well as the bacterial surface. Eap plays a complementary role together with FnBP, in the internalization and long time persistence of *S. aureus* within eukaryotic cells. It was found to be a key component of the novel internalization pathway that works either in parallel with, or in addition to, the FnBP dependent internalization pathway [41].

### Sec-independent Ess secretion pathway/ Type VII secretion system, T7SS

Many Gram-positive bacterial species, including *S. aureus*, secrete exotoxins or virulence factors across the membrane, through signal peptides or the Sec translocon. A Sec-independent translocation of these factors has also been reported in Gram-positive bacteria. Human *S. aureus* has been shown to secret the ESAT-6-like secretory proteins EsxA and EsxB. The genes encoding these proteins cluster in the genome as an operon together with several additional genes to form a secretion system, known as the type 7 secretion system (T7SS) that is involved in bacterial pathogenicity [42, 43]. The *S. aureus* isolate ILRI_Eymole1/1 possessed genes encoding proteins related to T7SS; which were also present in all other CC30 *S. aureus* genomes. These encoded the secretory antigen precursor SsaA (CEH25002), ESAT-6/Esx family secreted protein EsxA/YukE (CEH25003), putative secretion accessory protein EsaA/YueB (CEH25004), putative secretion system component EssA (CEH25005), putative secretion accessory protein EsaB/YukD (CEH25006), putative secretion system component EssB/YukC (CEH25007), and a FtsK/SpoIIIE family protein, together with putative secretion system component EssC/YukA (CEH25008).

### Isolate specific genes

Out of the total 85 isolate specific genes encoded by *S. aureus* ILRI_Eymole1/1, 79 genes (92.94 %) were clustered into six large insertions. Four insertions were putative bacteriophages comprising four complete phages with sizes of 52.5 kb, 30 kb, 60.3 kb and 58.8 kb, respectively. All phage sequences possessed *attL* and *attR* integration sequences at the forward and reverse ends. Superantigen pathogenicity islands (SaPI) are mobile genetic elements in Gram-positive bacteria including *S. aureus* that carry genes associated with superantigens, virulence, resistance and metabolic functions; also named as *S. aureus* pathogenicity islands ‘SaPI’. These are known for their strong association with temperate phages and result in high transfer frequencies [44]. Two insertions constituted complete SaPI islands (SaPIcam1 and SaPIcam2) at positions 426,323-443,273 and 758,187-774,130. These were confirmed by the identification of forward and reverse sequences at the 5′–3′ ends of previously characterized SaPIs, namely SaPIbov and SaPImw2 [45]. SaPIcam1 and SaPIcam2 both had integrase and terminase encoding genes at their termini. SaPIcam1 also possessed an HTH-type transcriptional regulator LrpC adjacent to 3′ end. The SaPIcam2 contained a candidate superantigen *tst* gene (toxic shock syndrome toxin 1 TSST-1, as part of variable gene content) located adjacent to the 3′ end. The ‘SaPI2’ island in the CC30 isolates encodes a TSST-1 gene, and these have a clonal association with CC30 nasal infective and bacteremia causing isolates [36, 46]. Among *S. aureus* CC30 isolates analyzed in this study, only ILRI_Eymole1/1, EMRSA16, A017934_97, Btn1260 and MN8 genomes contained ‘SaPI2’, encoding the *tst* gene. All other CC30 genomes possessed a ‘SaPI1’ island, and therefore encoded Ear, a secretory protein, at the 3′ end. The presence of the high level of isolate specific genes (92.94 %) in these phage insertions and the SaPI islands strongly suggests the acquisition of these genes through lateral gene transfer from either phages or heterologous bacterial species harboring these insertions.

### Phylogeny using polymorphic set of core genes

**Fig. 5:**
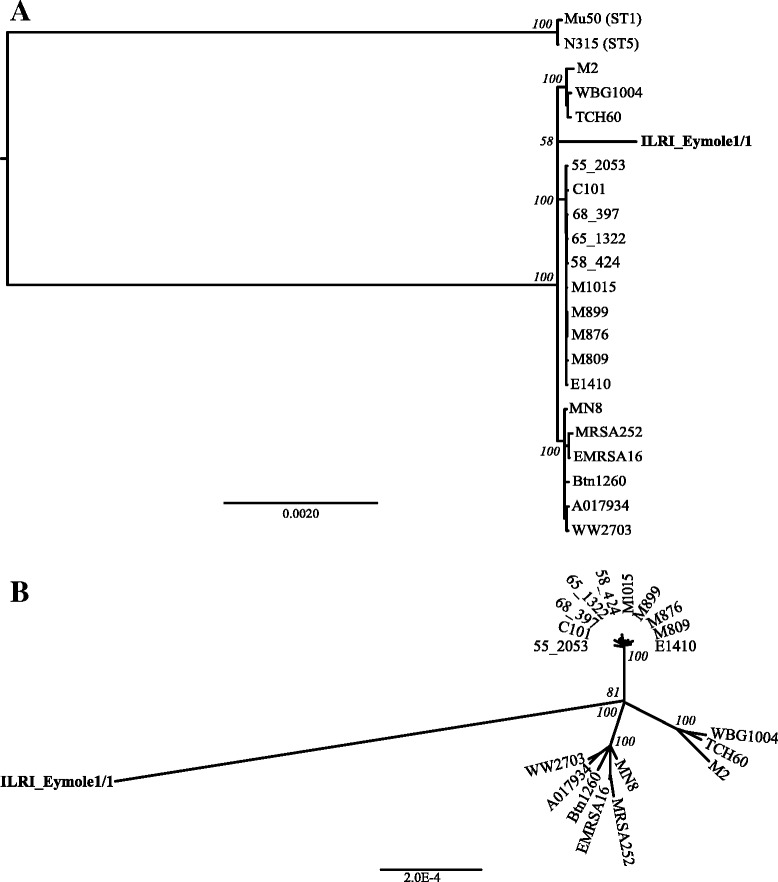
**a** Maximum likelihood tree of the concatenated sequence of selected 283 core genes in 20 CC30 *S. aureus* isolates; one ST1 and one ST5 *S. aureus* isolate Mu50 and N315 respectively. General Time Reverse model was used with 100 bootstrap replications. The bootstrap values are represented above the nodes. ST1 and ST5 are out grouped. **b** Maximum likelihood unrooted tree of 20 CC30 *S. aureus* isolates using set of 283 core genes. General Time Reverse model and 100 bootstrap replications were used. The values indicated are the bootstrap values

We determined the phylogenetic relationship among the isolates using a stringently defined set of 283 core genes that were shared among the CC30 isolates. Two *S. aureus* genomes from ST1 and ST5 were also included in this analysis as outgroups. The core genes were defined among these 22 *S. aureus* genomes using the criteria of (identity > = 95 % and < 100 %), (e-value < 0.00005) and (alignment length > = 90 %). Duplicate copies of genes were filtered out, resulting in the final total of 283 core genes. Multiple sequence alignment of the concatenated sequences of these genes was performed using the Mugsy aligner [47], generating an alignment comprising 316,359 nucleotides from each isolate. We estimated a maximum likelihood phylogeny using PhyML v. 3.0 [48]. The General Time-Reversible (GTR) model was used, where the base frequencies and the relative substitution rates between them were estimated by maximizing the likelihood of the phylogeny. For estimating the tree topology both nearest neighbor interchange and subtree pruning and regrafting methods were used. One hundred bootstrap replicates were run (Fig. 5a and b).

In both rooted and unrooted trees (Fig. 5a and b), the human CC30 isolates group in three clusters, in agreement with a published study [36]. The camel *S. aureus* isolate ILRI_Eymole1/1 clusters in the CC30 (Fig. 5a), but is genetically distant from human CC30 *S. aureus* isolates (Fig. 5b).

## Conclusion

Here we report the first genome of a *S. aureus* isolated from *Camelus dromedarius**.* Our analysis shows that a high proportion of isolate-specific genes were located in putative phage insertions and SaPI islands in the camel isolate clearly distinguished it from human isolates. The analysis based on a polymorphic set of core genes clearly shows that the camel *S .aureus* isolate belongs to ST30 but this isolate has greater genetic difference when compared to human isolates. Therefore, we consider the likelihood of exchange between camel and human populations low. However, this is the complete genome of a single *S. aureus* from a camel. The analysis of additional *S. aureus* isolates from camels and humans living in the same area, followed by a detailed comparative and phylogenetic analysis will underpin improved understanding of host adaptation and zoonotic potential.

## Additional files

Additional file 1: Table S1. Associated MIGS record. (DOC 73 kb) Additional file 2: Table S2. The 16S rRNA sequences of the type strains of genus *Staphylococcus* used in phylogenetic tree (Fig. 1). (DOC 47 kb) Additional file 3: Table S3. General genomic features of twenty CC30 *S. aureus* genomes. (DOC 53 kb) Additional file 4:
**Supplementary data.** (DOCX 143 kb)

## Abbreviations

CC30: Clonal Complex 30
MRSA: Methicillin Resistance *Staphylococcus aureus*
MSSA: Methicillin Susceptible/Sensitive *Staphylococcus aureus*
