# Supplementary material for: Complete genome sequence of Staphylococcus aureus, strain ILRI_Eymole1/1, isolated from a Kenyan dromedary camel
Source: Stand Genomic Sci. 2015 Nov 20;10:109. doi: 10.1186/s40793-015-0098-6 (PMC4654806; doi:10.1186/s40793-015-0098-6)
Supplement: Additional file 1: Table S1. — Associated MIGS record. (DOC 73 kb) [file 40793_2015_98_MOESM1_ESM.doc]

**Table S1.** Associated MIGS record

| **MIGS-ID** | field name | description |
| --- | --- | --- |
| **MIGS-1** | Submit to INSDC/Trace archives | [LN626917](http://www.ncbi.nlm.nih.gov/nuccore/LN626917) |
| **1.1** | PID | [PRJEB6577](http://www.ncbi.nlm.nih.gov/bioproject/PRJEB6577) |
| **1.2** | Trace Archive |  |
| **MIGS-2** | MIGS CHECK LIST TYPE |  |
| **MIGS-3** | Project Name |  |
| **MIGS-4** | Geographic Location | Kenya |
| **4.1** | Latitude | 3.916667 |
| **4.2** | Longitude | 41.833333 |
| **4.3** | Depth |  |
| **4.4** | Altitude | 220 |
| **MIGS-5** | Time of Sample collection | 02-01-2004 |
| **MIGS-6** | Habitat (EnvO) | Nasopharyngeal microflora |
| **6.1** | temperature | 37°C |
| **6.2** | pH | 7.0 |
| **6.3** | Salinity | 1 to 2.5M NaCl |
| **6.4** | chlorophyll |  |
| **6.5** | conductivity |  |
|
| **6.6** | light intensity |  |
| **6.7** | dissolved organic carbon (DOC) |  |
| **6.8** | current |  |
| **6.9** | atmospheric data |  |
| **6.10** | density |  |
| **6.11** | alkalinity |  |
| **6.12** | dissolved oxygen |  |
| **6.13** | particulate organic carbon (POC) |  |
| **6.14** | phosphate |  |
| **6.15** | nitrate |  |
| **6.16** | sulfates |  |
| **6.17** | sulfides |  |
| **6.18** | primary production |  |
| **MIGS-7** | Subspecific genetic lineage |  |
| **MIGS-9** | Number of replicons |  |
| **MIGS-10** | Extrachromosomal elements |  |
| **MIGS-11** | Estimated Size |  |
| **MIGS-12** | Reference for biomaterial or Genome report |  |
| **MIGS-13** | Source material identifiers |  |
| **MIGS-14** | Known Pathogenicity |  |
|
| **MIGS-15** | Biotic Relationship | Free living |
| **MIGS-16** | Specific Host |  |
| **MIGS-17** | Host specificity or range (taxid) |  |
| **MIGS-18** | Health status of Host | Respiratory disease |
| **MIGS-19** | Trophic Level |  |
| **MIGS-22** | Relationship to Oxygen | Facultative |
| **MIGS-23** | Isolation and Growth conditions | PureLink™ Genomic DNA Mini Kit (Invitrogen, USA), Liquid Brain heart medium (Carl Roth, Germany) |
| **MIGS-27** | Nucleic acid preparation |  |
| **MIGS-28** | Library construction | Paired end |
| **28.1** | Library size | Read size: 300bp, Insert-size: 550 bp |
| **28.2** | Number of reads | 1,154,246 |
| **28.3** | vector |  |
| **MIGS-29** | Sequencing method | Illumina GA-II |
| **MIGS-30** | Assembly | *De novo* assembly |
| **30.1** | Assembly method | MIRA v 4.0 |
| **30.2** | estimated error rate |  |
| **30.3** | method of calculation |  |
| **MIGS-31** | Finishing strategy |  |
| **31.1** | Status | Finished |
| **31.2** | coverage | 109X |
| **31.3** | contigs | 1 complete chromosome |
| **MIGS-32** | Relevant SOPs |  |
| **MIGS-33** | Relevant e-resources |  |
