# Supplementary material for: Complete genome sequence of Staphylococcus aureus, strain ILRI_Eymole1/1, isolated from a Kenyan dromedary camel
Source: Stand Genomic Sci. 2015 Nov 20;10:109. doi: 10.1186/s40793-015-0098-6 (PMC4654806; doi:10.1186/s40793-015-0098-6)
Supplement: Additional file 2: Table S2. — The 16S rRNA sequences of the type strains of genus Staphylococcus used in phylogenetic tree (Fig. 1). (DOC 47 kb) [file 40793_2015_98_MOESM2_ESM.doc]

**Table S2.** The 16S rRNA sequences of the type strains of genus [*Staphylococcus*](http://dx.doi.org/10.1601/nm.5230) used in phylogenetic tree (Figure 1)

| **Organism** | **GeneBank accession number** |
| --- | --- |
| [*S. simiae*](http://dx.doi.org/10.1601/nm.9586) | [AY727530](http://www.ncbi.nlm.nih.gov/nuccore/AY727530) |
| [*S. epidermidis*](http://dx.doi.org/10.1601/nm.5246) | [D83363](http://www.ncbi.nlm.nih.gov/nuccore/D83363) |
| [*S. saccharolyticus*](http://dx.doi.org/10.1601/nm.5267) | [L37602](http://www.ncbi.nlm.nih.gov/nuccore/L37602) |
| [*S. capitis*](http://dx.doi.org/10.1601/nm.11044) | [L37599](http://www.ncbi.nlm.nih.gov/nuccore/L37599) |
| [*S. caprae*](http://dx.doi.org/10.1601/nm.5237) | [AB009935](http://www.ncbi.nlm.nih.gov/nuccore/AB009935) |
| [*S. pasteuri*](http://dx.doi.org/10.1601/nm.5264) | [AB009944](http://www.ncbi.nlm.nih.gov/nuccore/AB009944) |
| [*S. warneri*](http://dx.doi.org/10.1601/nm.5281) | [L37603](http://www.ncbi.nlm.nih.gov/nuccore/L37603) |
| [*S. kloosii*](http://dx.doi.org/10.1601/nm.5258) | [AB009940](http://www.ncbi.nlm.nih.gov/nuccore/AB009940) |
| [*S. xylosus*](http://dx.doi.org/10.1601/nm.5282) | [D83374](http://www.ncbi.nlm.nih.gov/nuccore/D83374) |
| [*S. arlettae*](http://dx.doi.org/10.1601/nm.5233) | [AB009933](http://www.ncbi.nlm.nih.gov/nuccore/AB009933) |
| [*S. gallinarum*](http://dx.doi.org/10.1601/nm.5251) | [D83366](http://www.ncbi.nlm.nih.gov/nuccore/D83366) |
| [*S. microti*](http://dx.doi.org/10.1601/nm.17806) | [EU888120](http://www.ncbi.nlm.nih.gov/nuccore/EU888120) |
| [*S. rostri*](http://dx.doi.org/10.1601/nm.20126) | [FM242137](http://www.ncbi.nlm.nih.gov/nuccore/FM242137) |
| [*S. muscae*](http://dx.doi.org/10.1601/nm.5262) | [FR733703](http://www.ncbi.nlm.nih.gov/nuccore/FR733703) |
| [*S. hyicus*](http://dx.doi.org/10.1601/nm.11050) | [D83368](http://www.ncbi.nlm.nih.gov/nuccore/D83368) |
| [*S. lutrae*](http://dx.doi.org/10.1601/nm.5261) | [X84731](http://www.ncbi.nlm.nih.gov/nuccore/X84731) |
| [*S. delphini*](http://dx.doi.org/10.1601/nm.5245) | [AB009938](http://www.ncbi.nlm.nih.gov/nuccore/AB009938) |
| [*S. intermedius*](http://dx.doi.org/10.1601/nm.7084) | [D83369](http://www.ncbi.nlm.nih.gov/nuccore/D83369) |
| [*S. pseudintermedius*](http://dx.doi.org/10.1601/nm.9497) | [AJ780976](http://www.ncbi.nlm.nih.gov/nuccore/AJ780976) |
| [*S. carnosus*](http://dx.doi.org/10.1601/nm.11045) | [AB009934](http://www.ncbi.nlm.nih.gov/nuccore/AB009934) |
| [*S. piscifermentans*](http://dx.doi.org/10.1601/nm.5265) | [Y15754](http://www.ncbi.nlm.nih.gov/nuccore/Y15754) |
| [*B. subtilis*](http://dx.doi.org/10.1601/nm.10618) [DSM10](http://doi.org/10.1601/strainfinder?urlappend=%3Fid%3DDSM10) | [AJ276351](http://www.ncbi.nlm.nih.gov/nuccore/AJ276351) |
