# Supplementary material for: Complete genome sequence of Staphylococcus aureus, strain ILRI_Eymole1/1, isolated from a Kenyan dromedary camel
Source: Stand Genomic Sci. 2015 Nov 20;10:109. doi: 10.1186/s40793-015-0098-6 (PMC4654806; doi:10.1186/s40793-015-0098-6)
Supplement: Additional file 3: Table S3. — General genomic features of twenty CC30 S. aureus genomes. (DOC 53 kb) [file 40793_2015_98_MOESM3_ESM.doc]

***Table S3.*** *General genomic features of twenty CC30* [*S. aureus*](http://dx.doi.org/10.1601/nm.6874) *genomes*

| **CC30** [***S. aureus***](http://dx.doi.org/10.1601/nm.6874) **isolates** | **GenBank accession number** | **Strain type (ST)** | **Genome Status** | **Genome size (bp)*** | **GC%** | **rRNA genes** | **tRNA genes** |
| --- | --- | --- | --- | --- | --- | --- | --- |
| ILRI_Eymole1/1 | LN626917 | ST30 | Complete | 2,874,302 | 32.88 | 16 | 60 |
| 55/2053 | [NC_022113](http://www.ncbi.nlm.nih.gov/nuccore/NC_022113) | ST30 | Complete | 2,756,919 | 32.81 | 12 | 52 |
| 58_424 | NZ_ACUT00000000 | ST30 | 23 Scaffolds | 2,798,502 | 32.77 | 12 | 56 |
| 65_1322 | NZ_ACJS00000000 | ST30 | 15 Scaffolds | 2,777,288 | 32.75 | 4 | 56 |
| 68_397 | NZ_ACJT00000000 | ST30 | 23 Scaffolds | 2,767,646 | 32.76 | 4 | 52 |
| M1015 | NZ_ACST00000000 | ST30 | 20 Scaffolds | 2,764,225 | 32.76 | 4 | 56 |
| M809 | NZ_ACUS00000000 | ST431 | 27 Scaffolds | 2,770,328 | 32.72 | 4 | 40 |
| C101 | NZ_ACSP00000000 | ST30 | 21 Scaffolds | 2,781,680 | 32.74 | 4 | 56 |
| M899 | NZ_ACSU00000000 | ST30 | 18 Scaffolds | 2,768,587 | 32.75 | 4 | 56 |
| M876 | NZ_ACJV00000000 | ST30 | 16 Scaffolds | 2,762,707 | 32.76 | 4 | 56 |
| E1410 | NZ_ACJU00000000 | ST30 | 17 Scaffolds | 2,810,476 | 32.79 | 4 | 56 |
| MRSA252 | [NC_002952](http://www.ncbi.nlm.nih.gov/nuccore/NC_002952) | ST36 | Complete | 2,902,619 | 32.81 | 16 | 60 |
| EMRSA16 | NZ_ADAT00000000 | ST36 | 26 Scaffolds | 2,879,199 | 32.71 | 12 | 56 |
| A017934_97 | NZ_ACYP00000000 | ST30 | 20 Scaffolds | 2,801,823 | 32.69 | 16 | 59 |
| WW2703_97 | NZ_ACSW00000000 | ST30 | 36 Scaffolds | 2,743,582 | 32.70 | 4 | 47 |
| Btn1260 | NZ_ACUU00000000 | ST30 | 17 Scaffolds | 2,780,198 | 32.70 | 13 | 56 |
| MN8 | NZ_CM000952.1 | ST30 | Complete | 2,882,664 | 32.75 | 14 | 56 |
| MRSA-M2 | NZ_AMTC00000000 | ST30 | 133 Contigs | 2,829,186 | 32.66 | 5 | 44 |
| [WBG10049](http://doi.org/10.1601/strainfinder?urlappend=%3Fid%3DWBG10049) | NZ_ACSV00000000 | ST30 | 12 Scaffolds | 2,777,174 | 32.69 | 4 | 55 |
| TCH60 | [NC_017342](http://www.ncbi.nlm.nih.gov/nuccore/NC_017342) | ST30 | Complete | 2,802,675 | 32.87 | 19 | 59 |

**‘*’:** The genome sizes of draft genomes (sixteen in number) are total sum of the sizes of all contigs/scaffolds in each genome. Moreover, the stretches of Ns are also excluded from genome size.
