# Supplementary material for: Complete genome sequence of Staphylococcus aureus, strain ILRI_Eymole1/1, isolated from a Kenyan dromedary camel
Source: Stand Genomic Sci. 2015 Nov 20;10:109. doi: 10.1186/s40793-015-0098-6 (PMC4654806; doi:10.1186/s40793-015-0098-6)
Supplement: Additional file 4: — Supplementary data. (DOCX 143 kb) [file 40793_2015_98_MOESM4_ESM.docx]

**Supplementary data**

**Custom scripts in Perl language that were used to perform the core genome analysis of CC30 *S. aureus* isolates, in this manuscript:**

### Author: Saima Zubair

### Email: [saimazubair.mail@gmail.com](mailto:saimazubair.mail@gmail.com)

### First of all, the amino acid sequences (of protein coding genes) containing files for all CC30 isolates were concatenated to get a single file containing all sequences in one file. This file was named as CC30-all.faa, and below commands were run to do blastp comparison of genes;

formatdb -p T -i CC30-all.faa

blastall -p blastp -d CC30-all.faa -i ILRIEymole1.faa -m8 -F F -o ILRIEymole1-VS-allCC30

### Below Perl script was used to filter the blast output on the criteria of greater than or equal to 80% identity and less than 0.00005 E-value.

#! /usr/bin/perl

open (IN," ILRIEymole1-VS-allCC30.txt") or die "cannot open file, $!\n";

open (OUT,"> ILRIEymole1-VS-allCC30-ep");

while ($line = <IN>)

{

@elements = split ("\t", $line);

if (@elements[2]>=80 && @elements[10]<0.00005)

{

print OUT $line;

}

}

close IN;

close OUT;

print "Filtering (Percent identity AND E-value) done in file:", $i+1, "\n";

print "Filtering DONE\n";

### Below Perl script was used to count the length of genes for ‘ILRI_Eymole1/1’ genome. Output was saved in a file in form of two columns, first column: the gene ID, and the second column: length of that gene.

$char ="\n";

open (IN," ILRIEymole1.faa") or die "cannot open file, $!\n";

open (OUT,"> ILRIEymole1-GL");

$residue = 0;

while ($line = <IN>)

{

if ($line =~/>/g) {

$IndexValue =index($line,$char);

$ID=substr($line,1,$IndexValue-1);

print OUT "$ID\t",$residue*3,"\n";

$residue=0;

}

else {

while ($line =~ /[A-Z]/ig) {$residue++;}

}

}

print OUT "$ID\t",$residue*3,"\n";

close IN;

close OUT;

print "Residues length calculated in file. \n";

### Below Perl script was used to insert the gene length calculated from above script, into the blast-output that was filtered according to E-value and percent identity. It further calculated the Alignment-length%.

#! /usr/bin/perl

open (IN2,"ILRIEymole1-VS-allCC30-ep") or die "cannot open file, $!\n";

open (OUT1,">ILRIEymole1-VS-allCC30-epGL");

while ($line2 = <IN2>)

{

my @arry2 = split ("\t", $line2);

my @column2 = grep(s/\s*$//g, @arry2);

open (IN1, "ILRIEymole1-GL") or die "cannot open file, $!\n";

while ($line1 = <IN1>)

{

my @arry1 = split ("\t", $line1);

my @column1 = grep(s/\s*$//g, @arry1);

if (@column1[0] eq @column2[0])

{

$round = sprintf("%.3f", (@column2[3] * 300) / @column1[1]);

print OUT1 @column2[0],"\t", @column2[1],"\t", @column2[2],"\t", @column2[3],"\t", @column2[4],"\t", @column2[5],"\t";

print OUT1 @column2[6],"\t", @column2[7],"\t", @column2[8],"\t", @column2[9],"\t", @column2[10],"\t", @column2[11],"\t", @column1[1],"\t";

print OUT1 $round, "\n";

last;

}

}

close IN1;

}

close IN2;

close OUT1;

print "file done =", $i+1, "\n";

### Below Perl script was used to extract genes with at least 50% alignment length.

#! /usr/bin/perl

open (IN,"ILRIEymole1-VS-allCC30-epGL") or die "cannot open file, $!\n";

open (OUT,">ILRIEymole1-VS-allCC30-epGL50");

while ($line = <IN>)

{

@elements = split ("\t", $line);

if (@elements[13]>=50)

{

print OUT $line;

}

}

close IN;

close OUT;

print "Genes with 50% coverage length extracted, in file:", $i+1, "\n";

### Below Perl script was used on above output, and clusters were identified that had genes common from 2 to 20 CC30 genomes. Further, the isolate specific genes in ILRI_Eymole1/1 were also identified.

#! /usr/bin/perl

$char ="\t";

open (IN,"ILRIEymole1-GL") or die "cannot open file, $!\n";

open (OUT,">ILRIEymole1-iSpecific");

open (OUT1,">ILRIEymole1-based-Clustering"); //Variable+core

while ($line = <IN>)

{

$IndexValue =index($line,$char);

$ID=substr($line,0,$IndexValue);

#print $IndexValue, "\n";

print $ID, "\n";

print OUT1

"WW2703_97=", $WW2703_97_var, "; ",

"WBG10049=", $WBG10049_var, "; ",

"TCH60=", $TCH60_var, "; ",

"MRSA252=", $MRSA252_var, "; ",

"MN8=", $MN8_var, "; ",

"M899=", $M899_var, "; ",

"M876=", $M876_var, "; ",

"M809=", $M809_var, "; ",

"MRSA-M2=", $MRSA-M2_var, "; ",

"M1015=", $M1015_var, "; ",

"EMRSA16=", $EMRSA16_var, "; ",

"E1410=", $E1410_var, "; ",

"C101=", $C101_var, "; ",

"Btn1260=", $Btn1260_var, "; ",

"A017934_97=", $A017934_97_var, "; ",

"68_397=", $s68_397_var, "; ",

"65_1322=", $s65_1322_var, "; ",

"58_524=", $s58_524_var, "; ",

"55_2053=", $s55_2053_var, "; ",

;

print OUT1 "CLUSTER-COUNT=", $sum, "\n\n\n";

$sum=0;

$WW2703_97_var=$WW2703_97_var=$WBG10049_var=$TCH60_var=$MRSA252_var=$MN8_var=$M899_var=$M876_var=$M809_var=$MRSA-M2_var=$M1015_var=$EMRSA16_var=$E1410_var=$C101_var=$Btn1260_var=$A017934_97_var=$s68_397_var=$s65_1322_var=$s58_524_var=$s55_2053_var=0;

open (IN1,"ILRIEymole1-VS-allCC30-epGL50") or die "cannot open present genes files";

$counter=0;

while($line1 = <IN1>)

{

if ($line1 =~/$ID\b/g)

{

$counter=1;

print OUT1 $line1;

$sum=$sum+1;

if ($line1 =~/WW2703_97:/g)

{

$WW2703_97_var++;

}

if ($line1 =~/WBG10049:/g)

{

$WBG10049_var++;

}

if ($line1 =~/TCH60:/g)

{

$TCH60_var++;

}

if ($line1 =~/MRSA252:/g)

{

$MRSA252_var++;

}

if ($line1 =~/MN8:/g)

{

$MN8_var++;

}

if ($line1 =~/M899:/g)

{

$M899_var++;

}

if ($line1 =~/M876:/g)

{

$M876_var++;

}

if ($line1 =~/M809:/g)

{

$M809_var++;

}

if ($line1 =~/M2:/g)

{

$MRSA-M2_var++;

}

if ($line1 =~/M1015:/g)

{

$M1015_var++;

}

if ($line1 =~/EMRSA16:/g)

{

$EMRSA16_var++;

}

if ($line1 =~/E1410:/g)

{

$E1410_var++;

}

if ($line1 =~/C101:/g)

{

$C101_var++;

}

if ($line1 =~/Btn1260:/g)

{

$Btn1260_var++;

}

if ($line1 =~/A017934_97:/g)

{

$A017934_97_var++;

}

if ($line1 =~/68_397:/g)

{

$s68_397_var++;

}

if ($line1 =~/65_1322:/g)

{

$s65_1322_var++;

}

if ($line1 =~/58_524:/g)

{

$s58_524_var++;

}

if ($line1 =~/55_2053:/g)

{

$s55_2053_var++;

}

}

}

if ($counter ==0)

{

print OUT $line;

}

}

close IN1;

close IN;

close OUT;

print "Process finished","\n";

### Below commands were applied on above output to separate the Core clusters (in all 20 isolates), from variable/shared clusters (in 2 to 19 isolates).

grep 'CLUSTER-COUNT' ILRIEymole1-based-Clustering | awk '!/=0/' ILRIEymole1-based-Clustering > CORE-CLUSTERS

awk '!/COUNT=0/' ILRIEymole1-based-Clustering > VARIABLE+CORE

grep -1 '=0' VARIABLE-and-CORE > VARIABLE-CLUSTERS-ILRIEymole1

######## Below command was run to save ILRIEymole1's core genes in separate file.

######## grep -1 'CLUSTER-COUNT' CORE-CLUSTERS > ILRIEymole1-core-CC30

### Below Perl script was run to save the core genes of each isolate in separate files.

$char ="\t";

open (IN,"ILRIEymole1-core-CC30") or die "cannot open file, $!\n";

open (OUT1,">WW2703_97-core-CC30");

open (OUT2,">WBG10049-core-CC30");

open (OUT3,">TCH60-core-CC30");

open (OUT4,">MRSA252-core-CC30");

open (OUT5,">MN8-core-CC30");

open (OUT6,">M899-core-CC30");

open (OUT7,">M876-core-CC30");

open (OUT8,">M809-core-CC30");

open (OUT9,">MRSA-M2-core-CC30");

open (OUT10,">M1015-core-CC30");

open (OUT11,">EMRSA16-core-CC30");

open (OUT12,">E1410-core-CC30");

open (OUT13,">C101-core-CC30");

open (OUT14,">Btn1260-core-CC30");

open (OUT15,">A017934_97-core-CC30");

open (OUT16,">68_397-core-CC30");

open (OUT17,">65_1322-core-CC30");

open (OUT18,">58_524-core-CC30");

open (OUT19,">55_2053-core-CC30");

while ($line = <IN>)

{

while ($line =~/>/g)

{

$IndexValue =index($line,$char);

$ID=substr($line,1,$IndexValue-1);

print $ID, "\n";

open (IN1,"ILRIEymole1-based-Clustering") or die "cannot open present genes files";

while($line1 = <IN1>)

{

if ($line1 =~/$ID\b/g)

{

if ($line1 =~/WW2703_97:/g)

{

print OUT1 $line1;

}

if ($line1 =~/WBG10049:/g)

{

print OUT2 $line1;

}

if ($line1 =~/TCH60:/g)

{

print OUT3 $line1;

}

if ($line1 =~/MRSA252:/g)

{

print OUT4 $line1;

}

if ($line1 =~/MN8:/g)

{

print OUT5 $line1;

}

if ($line1 =~/M899:/g)

{

print OUT6 $line1;

}

if ($line1 =~/M876:/g)

{

print OUT7 $line1;

}

if ($line1 =~/M809:/g)

{

print OUT8 $line1;

}

if ($line1 =~/M2:/g)

{

print OUT9 $line1;

}

if ($line1 =~/M1015:/g)

{

print OUT10 $line1;

}

if ($line1 =~/EMRSA16:/g)

{

print OUT11 $line1;

}

if ($line1 =~/E1410:/g)

{

print OUT12 $line1;

}

if ($line1 =~/C101:/g)

{

print OUT13 $line1;

}

if ($line1 =~/Btn1260:/g)

{

print OUT14 $line1;

}

if ($line1 =~/A017934_97:/g)

{

print OUT15 $line1;

}

if ($line1 =~/68_397:/g)

{

print OUT16 $line1;

}

if ($line1 =~/65_1322:/g)

{

print OUT17 $line1;

}

if ($line1 =~/58_524:/g)

{

print OUT18 $line1;

}

if ($line1 =~/55_2053:/g)

{

print OUT19 $line1;

}

}

}

}

}

close IN1;

close IN;

close OUT1;

close OUT2;

close OUT3;

close OUT4;

close OUT5;

close OUT6;

close OUT7;

close OUT8;

close OUT9;

close OUT10;

close OUT11;

close OUT12;

close OUT13;

close OUT14;

close OUT15;

close OUT16;

close OUT17;

close OUT18;

close OUT19;

print "Process finished","\n";
